# Microbiological and Molecular Characterization of *Staphylococcus hominis* Isolates from Blood

**DOI:** 10.1371/journal.pone.0061161

**Published:** 2013-04-09

**Authors:** Soraya Mendoza-Olazarán, Rayo Morfin-Otero, Eduardo Rodríguez-Noriega, Jorge Llaca-Díaz, Samantha Flores-Treviño, Gloria Ma González-González, Licet Villarreal-Treviño, Elvira Garza-González

**Affiliations:** 1 Departamento de Microbiología, Facultad de Medicina, Universidad Autónoma de Nuevo León. Monterrey Nuevo León, México; 2 Hospital Civil de Guadalajara, Fray Antonio Alcalde, and Instituto de Patología Infecciosa y Experimental, Centro Universitario de Ciencias de la Salud, Universidad de Guadalajara. Guadalajara, Jalisco México; 3 Departamento de Patología Clínica, Hospital Universitario Dr. José Eleuterio González, Universidad Autónoma de Nuevo León. Monterrey Nuevo León, México; 4 Departamento de Microbiología e Inmunología, Facultad de Ciencias Biológicas, Universidad Autónoma de Nuevo León. Monterrey Nuevo León, México; 5 Servicio de Gastroenterología Universidad Autónoma de Nuevo León. Monterrey Nuevo León, México; Rockefeller University, United States of America

## Abstract

**Background:**

Among Coagulase-Negative Staphylococci (CoNS), *Staphylococcus hominis* represents the third most common organism recoverable from the blood of immunocompromised patients. The aim of this study was to characterize biofilm formation, antibiotic resistance, define the SCC*mec* (Staphylococcal Chromosomal Cassette *mec*) type, and genetic relatedness of clinical *S. hominis* isolates.

**Methodology:**

*S. hominis* blood isolates (n = 21) were screened for biofilm formation using crystal violet staining. Methicillin resistance was evaluated using the cefoxitin disk test and the *mec*A gene was detected by PCR. Antibiotic resistance was determined by the broth microdilution method. Genetic relatedness was determined by pulsed-field gel electrophoresis (PFGE) and SCC*mec* typed by multiplex PCR using two different methodologies described for *Staphylococcus aureus*.

**Results:**

Of the *S. hominis* isolates screened, 47.6% (10/21) were categorized as strong biofilm producers and 23.8% (5/21) as weak producers. Furthermore, 81% (17/21) of the isolates were methicillin resistant and *mec*A gene carriers. Resistance to ampicillin, erythromycin, and trimethoprim was observed in >70% of isolates screened. Each isolate showed a different PFGE macrorestriction pattern with similarity ranging between 0–95%. Among *mec*A-positive isolates, 14 (82%) harbored a non-typeable SCC*mec* type: eight isolates were not positive for any *ccr* complex; four contained the *mec* complex A *ccrAB1* and *ccrC*, one isolate contained *mec* complex A, *ccrAB4* and *ccrC*, and one isolate contained the *mec* complex A, *ccrAB1*, *ccrAB4*, and *ccrC*. Two isolates harbored the association: *mec* complex A and *ccrAB1*. Only one strain was typeable as SCC*mec* III.

**Conclusions:**

The *S. hominis* isolates analyzed were variable biofilm producers had a high prevalence of methicillin resistance and resistance to other antibiotics, and high genetic diversity. The results of this study strongly suggested that *S. hominis* isolates harbor new SCC*mec* structural elements and might be reservoirs of *ccrC1* in addition to *ccrAB1* and *mec* complex A.

## Introduction

Coagulase-negative staphylococci (CoNS) represent a group of opportunistic microorganisms commonly associated with infections of immunocompromised patients [1]. Among CoNS, *Staphylococcus hominis* is one of the three most frequently identified isolates recoverable from the blood of neonates and immunosuppressed patients [2], [3] and has been associated as a causal agent of bacteremia, septicemia, and endocarditis [3]–[7]. Nosocomial infections caused by CoNS are associated with the use of indwelling medical devices in combination with biofilm-forming potential of respective isolates [8]–[10]. However, among the CoNS, *S. hominis* strains are not typically categorized as a major biofilm producers [9], [11]. It has been reported that some *S. hominis* isolates are resistant to methicillin that is conferred by protein PBP2a encoded by the *mecA* gene that resides within a mobile genetic element called the Staphylococcal Cassette Chromosome *mec* (SCC*mec*) [12]. At present, eleven SCC*mec* types (I–XI) of have been assigned for *S. aureus* based on the classes of the *mec* gene complex (A–E) and the *ccr* gene complex (1–8) (http://www.sccmec.org/Pages/SCC_TypesEN.html). Some studies have reported that SCC*mec* elements are more diverse in methicillin-resistant CoNS, with new variants of *ccr* genes continually being identified [13]–[20].

A recent molecular epidemiologic study of *S. hominis* isolates conducted by Bouchami *et al.*, 2011 demonstrated low clonality between isolates and the identification of isolates harboring the SCC*mec* type VI, VIII, and the new SCC*mec* type composed of *mec* complex A (in combination with *ccrAB1*). In addition, some isolates harbored the non-typeable SCC*mec* in the absence of the *ccr* complex and others expressed two *ccr* types (in the same isolate). Additionally, *ccrB1* and *ccrB4* were identified in *mecA*-negative and *mecA*-positive isolates with high nucleotide sequence homology to genes present in *S. aureus* isolates expressing SCC*mec* I, VI, or VIII, respectively (>95%) [21].

In agreement with a report by Hanssen *et al.*, 2004 staphylococcal strains from the same geographical region possess identical *ccr* genes that differ from sequences of strains from other regions. There is evidence of horizontal SCC*mec* gene transfer between CoNS and *S. aureus* [22], [23]; therefore, characterization of SCC*mec* of *S. hominis* can provide useful information regarding the evolution and mobilization of this element from this species. The aim of this study was to characterize biofilm formation potential, antibiotic resistance, SCC*mec* type, and genetic relatedness of 21 *S. hominis* clinical isolates obtained from blood cultures.

## Materials and Methods

### Ethics Statement

This study was performed with the approval of the Local Ethics Committee of the School of Medicine of the Universidad Autónoma de Nuevo León (Approval MB11-006). Informed consent was not required since bacterial isolates were the subject of this study. Isolates, not human beings were studied. Thus, informed consent was not required by the local Ethics Committee.

### Clinical isolates

*S. hominis* clinical isolates (n = 21) were collected between January 2006 and December 2011 from blood cultures from two hospitals in Mexico: Hospital Civil Fray Antonio Alcalde and Hospital Universitario Dr. José Eleuterio González. All isolates were causative agents of Laboratory-Confirmed Bloodstream Infection (LCBI) according to CDC criteria (http://www.cdc.gov/nhsn/pdfs/pscmanual/17pscnosinfdef_current.pdf). Isolates examined met at least one of the following criteria: a) Patient had a recognized pathogen cultured from two or more blood cultures and organisms cultured from blood were not related to an infection at another site, b) Ppatient had at least one of the following signs or symptoms: fever (>38°C), chills, or hypotension and positive laboratory results not related to an infection at another site. Isolates were kept frozen in *Brucella* broth containing 15% glycerol at −70°C. Only one isolate per patient was included in this study.

#### Identification of isolates

Isolates were identified at the species level using API Staph galleries (bioMérieux, Inc., Durham, NC) according to the manufacturer's instructions. Species identification was confirmed by partial sequencing of the 16S rRNA and the *tuf* genes as previously described [24]. Sequencing was performed at the Instituto de Biotecnología, Universidad Nacional Autónoma de México. DNA sequences were compared to gene sequences at the National Center for Biotechnology Information (NCBI) GenBank using the BLAST algorithm (http://www.ncbi.nlm.nih.gov/BLAST).

#### Phenotypic biofilm assay

Semi quantitative determination of biofilm formation was performed by crystal violet staining as previously described [10], [25]. All isolates were tested in quadruplicate in two different experiments conducted on different days. These assays were conducted on polystyrene 96-well flat bottom, untreated plates with a low evaporation lid. Biofilm-forming capacity of all isolates was tested under two different growth conditions: in trypticase soy broth (TSB) supplemented with 1% glucose (TSBglu) or in TSB supplemented with 3% NaCl (TSB NaCl). Briefly, biofilm samples stained with crystal violet were dissolved in an ethanol–acetone mixture (70∶30). The optical density of these solutions was subsequently measured at 550 nm. To simplify the data we used the ordinal classification for the level of biofilm production proposed by Christensen *et al.* Isolates with optical densities OD ≥0.25 were considered strong biofilm producers and isolates with optical densities between 0.15 and 0.24 were considered weak biofilm producers.

*Staphylococcus saprophyticus* ATCC 15305 (biofilm producer) and *S. hominis* ATCC 27844 (biofilm non-producer) were used as control organisms.

#### Methicillin resistance and susceptibility testing

Methicillin resistance was evaluated using the cefoxitin disk test and the *mec*A gene was detected by polymerase chain reaction (PCR) [26], [27]. During the cefoxitin disk evaluation, isolates were considered resistant if measurements were ≥24 mm and susceptible if measurements were ≤25 mm [27]. Susceptibility testing was performed using the broth microdilution method as recommended by the Clinical and Laboratory Standards Institute (CLSI) [27]. The antibiotics tested were penicillin, ampicillin, amoxicillin-clavulanic acid, cefotaxime, vancomycin, daptomycin, gentamicin, erythromycin, tetracycline, ciprofloxacin, nitrofurantoin, trimethoprim, chloramphenicol, rifampin, and linezolid (Sigma Aldrich, Toluca, Mexico).

#### SCC*mec* and PFGE typing

SCC*mec*, *ccr*, and *mec* class typing was performed as previously described by Zhang *et al.* [26] and Kondo *et al.* [28] with modification to three primers as previously described Ruppe *et al* [29]. All SCC*mec* typing experiments were performed in duplicate. As control strains we used for all PCR reactions isolates previously typed by Garza-González *et al.*, 2010: *Staphylococcus epidermidis* JC-5, JC-6, JC-28, JC-30, JC-488, JC-1439 and *Staphylococcus haemolyticus* JC-2165 [14], [30]. PFGE was performed as described for *S. aureus* [31] with modifications to the restriction enzymes used and running conditions were as previously described by Bouchami *et al.* [21]. *S. hominis* DNA samples were digested with the *Xho*I endonuclease and bands were separated using a CHEF-DRIII instrument (Bio-Rad Laboratories, Hercules, CA). Band patterns were generated by visual analysis using Labworks 4.5 software with 1% of tolerance. The similarity coefficients were generated from a similarity matrix calculated using the Jaccard coefficient (SPSS 20.0 software).

## Results

### Biofilm formation

By assay with TBSglu, 47.6% (10/21) of the *S. hominis* isolates were categorized as strong biofilm producers (defined by the cut-off values used in this study). Weak biofilm production was observed in 23.8% (5/21) of the isolates and 28.6% (6/21) were non-producers. Whereas by assay with TBS NaCl, 33.3% (7/21) were strong biofilm producers, 23.8% (5/21) weak producers, and 42.9 (9/21) non-producers (Table 1).

**Table 1:** Molecular and phenotypic characterization of *S. hominis* blood isolates.

Isolate	Biofilm^1^		FOX^2^	*mecA* 3	Zhang^4^		Kondo^4^		SCC*mec* type^5^	Resistance profile^6^												
	Glu	NaCl			*mec*	*ccr*	*mec*	*ccr*														
397	Strong	Strong	R	Pos	A	1+5	A	1+5	UT1	PEN,	AMP,	AUG,	GEN,	ERY,	TET,	TMP,	RIF					
501	Weak	Neg	S	Neg	Neg	Neg	Neg	Neg	Neg	PEN,	AMP,	AUG,	ERY,	CIP								
1786	Neg	Neg	R	Pos	B	Neg	B	Neg	UT	PEN,	AMP,	AUG,	CTX,	GEN,	ERY,	TET,	CIP,	NIT,	TMP,	CHL,	RIF	DAP*
8115	Neg	Neg	R	Pos	A	1+5	A	1+5	UT1	PEN,	AMP,	AUG,	ERY,	CIP,	TMP,	CHL						
8122	Neg	Neg	R	Pos	A	1+5	A	1+4+5	UT2	PEN,	AMP,	AUG,	ERY,	TET,	CIP,	TMP,	CHL					
8125	Neg	Neg	S	Neg	Neg	Neg	Neg	Neg	Neg	PEN,	AMP,	AUG,	ERY,	TMP								
8127	Weak	Neg	R	Pos	A	Neg	A	Neg	UT	PEN,	AMP,	AUG,	ERY,	TET,	TMP	DAP*						
8129	Weak	Weak	R	Pos	A	1	A	1	New	PEN,	AMP,	AUG,	GEN,	ERY,	TET							
8144	Neg	Neg	R	Pos	A	Neg	A	Neg	UT	PEN,	AMP,	AUG,	ERY,	TET,	CIP,	TMP						
8179	Neg	Neg	R	Pos	A	3	A	3	III	PEN,	AMP,	AUG,	TMP,	CHL	DAP*							
9241	Strong	Weak	S	Neg	Neg	Neg	Neg	Neg	Neg	ERY												
9989	Strong	Strong	R	Pos	A	1	A	1	New	PEN,	AMP,	AUG,	ERY,	CIP,	TMP,	CHL						
9994	Strong	Weak	R	Pos	A	5	A	4+5	UT3	PEN,	AMP,	AUG,	GEN,	ERY,	TMP,	CHL						
10866	Weak	Neg	R	Pos	A	Neg	A	Neg	UT	PEN,	AMP,	AUG,	ERY,	CIP,	TMP	DAP*						
11200	Strong	Weak	R	Pos	B	Neg	B	Neg	UT	PEN,	AMP,	AUG,	ERY,	TET,	CIP,	NIT,	TMP,	RIF	DAP*			
11477	Strong	Strong	S	Neg	Neg	Neg	Neg	Neg	Neg	PEN,	AMP,	AUG,	ERY	DAP*								
11621	Strong	Strong	R	Pos	A	Neg	A	Neg	UT	PEN,	AMP,	AUG,	ERY,	TMP,	CHL	DAP*						
11628	Strong	Strong	R	Pos	A	Neg	A	Neg	UT	PEN,	AMP,	AUG,	GEN,	ERY,	CIP,	TMP	DAP*					
11630	Strong	Strong	R	Pos	A	1+5	A	1+5	UT1	PEN,	AMP,	AUG,	GEN,	ERY,	TET,	CIP,	TMP					
11631	Strong	Strong	R	Pos	A	1+5	A	1+5	UT1	PEN,	AMP,	AUG,	CTX,	GEN,	ERY,	TET,	CIP,	TMP,	CHL,	RIF	DAP*	
11634	Weak	Weak	R	Pos	A	Neg	A	Neg	UT	PEN,	AMP,	AUG,	ERY	DAP*								

1 Neg: biofilm non-producer; Glu: media with 1% glucose; NaCl: media with 3% NaCl.

2 FOX: cefoxitin test; R: resistant; S: susceptible.

3 Pos: *mecA* gene present, Neg: *mecA* absent.

4 Neg: not amplified.

5 SCCmec type III was assigned for *S. aureus* according to http://www.sccmec.org/Pages/SCC_TypesEN.html.

Neg: not amplified, UT: untyped; UT1, UT2 and UT3 types are assigned in this study.

6 PEN: penicillin; AMP: ampicillin; AUG: amoxicillin-clavulanic acid; ERY: erythromycin; TMP: trimethoprim; CIP: ciprofloxacin; TET: tetracycline; CHL: chloramphenicol; GEN: gentamicin; RIF: rifampin; NIT: nitrofurantoin; CTX: cefotaxime; DAP: daptomycin.

* Non-susceptible.

All isolates were susceptible to vancomycin and linezolid.

### Methicillin resistance and susceptibility testing

Most isolates, 81% (17/21), showed methicillin resistance by the cefoxitin disk test, and all isolates tested positive for the *mec*A gene (Table 1). All *S. hominis* isolates were resistant to at least one of the non-β-lactam antibiotics tested. Resistance rates for penicillin, ampicillin, amoxicillin-clavulanic acid, erythromycin, trimethoprim, ciprofloxacin, tetracycline, chloramphenicol, gentamicin, rifampin, nitrofurantoin, and cefotaxime for all isolates were 95%, 95%, 95%, 95%, 76%, 52%, 43%, 38%, 33%, 19%, 10%, and 10%, respectively. Furthermore, 48% of isolates were daptomycin-non susceptible. None of the 21 isolates tested in this study were found to be resistant to vancomycin or linezolid. The 4 *mecA*-negative isolates showed resistance to at least one of the β-lactam antibiotics tested. No correlation was found between the level of biofilm production and the resistance phenotype.

### SCC*mec* and PFGE typing

A high frequency of *mec* complex class A (88.2%), *ccrAB1* (41.1%), and *ccrC* (35.3%) was observed among *mecA*-positive *S. hominis* isolates (Table 1).

Among the 17 *mecA*-positive isolates a high proportion were non-typeable (82%), eight were negative for *ccr* complex tested by both methods (UT); four isolates had a *mec* complex A*ccrAB1* and *ccrC* (UT1), one isolate had the *mec* complex A, *ccrAB4* and *ccrC* (UT2), and; one isolate had the *mec* complex A, *ccrAB1*, *ccrAB4* and *ccrC* (UT3). Two isolates carried association *mec* complex A and *ccrAB1*. One strain had SCC*mec* type III described for *S. aureus* (Table 1) (*mec* complex A, *ccr* 3, and isolate 8179).

**Figure 1:**
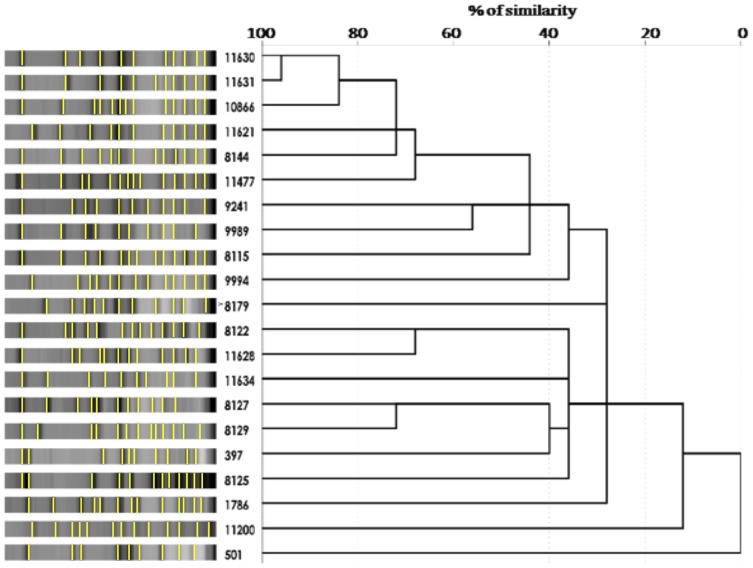
PFGE dendrogram of *S. hominis* isolates. Similarity coefficients were generated from a similarity matrix calculated with the Jaccard coefficient using SPSS 20.0 software.

PFGE analysis of *S. hominis* isolates identified 21 different restriction patterns with at least 3 band differences between each isolate (Figure 1). Although a 100% similarity was not observed between isolates, two isolates had 95% similarity (11630 and 11631) and were categorized as strong biofilm producers, *mec*A-positive, *mec* class A, *ccrAB1*+*ccrC*, and only differed in their susceptibility pattern.

## Discussion

Most studies examining the presence of SCC*mec* among CoNS isolates have included in their respective analyses few *S. hominis* clinical isolates recovered from catheters, the catheter insertion site, pus, wound secretions, cerebral spinal fluid, or blood [11], [13], [14], [21], [30], [32]–[34]. *S. hominis* comprises part of the normal flora colonizing the skin and mucous membranes of humans and may be found as a culture contaminant. However, detection of *S. hominis* is indicative of an infection and a probable causative agent of bacteremia. In this study, we analyzed 21 *S. hominis* clinical isolates recovered from blood and were causative agents of Laboratory-Confirmed Bloodstream Infection (LCBI) according to CDC criteria. To our knowledge, this is the first report characterizing *S. hominis* isolates identified as causative agents of bacteremia recovered from the blood at the microbiological and molecular level.

A significant observation associated with the *S. hominis* isolates studied was the ability of almost half of these strains (47.6%) to produce biofilm (since *S. hominis* is not known as a major biofilm producer) [9], [11]. This characteristic represents a significant virulence factor since biofilms facilitate bacterial adherence to biomedical surfaces (such as catheters), thereby facilitating their entrance into the bloodstream [8]. However, the polysaccharide or protein composition of *S. hominis* biofilms (or genes involved on its production) remains unknown to date.

Among the *mecA*-positive isolates (81%), nearly half were carriers of a putative new SCC*mec*. In addition, most expressed the *mec* gene class A, *ccr* type 1, and others *ccr* type 5. This combination of *mec-ccr* complexes has been reported in this bacterial species before [11], [14], [21], [32].

The *mec-ccr* complexes identified in this study were similar to those reported by Bouchami *et al.* that demonstrated that *S. hominis* could serve as a *mec-ccr* reservoir and also serve as a likely donor of *ccr*AB1 and *mec* complex A to other bacterial species. Unlike that study, we found a higher proportion of non-typeable isolates (82%) and isolates harboring *ccrC* (29%).

The data regarding SCC*mec* diversity in CoNS presented in this study may be biased due to the typing methodology used that was developed for *S. aureus*, therefore caution should be taken in the interpretation of these data. Therefore, a variety of non-typeable elements in CoNS may be simply an indication that *S. hominis* elements are different enough from those of *S. aureus* that the present typing methods can not be applied to this CoNS.

Data presented in this report also demonstrated that most isolates with new or untypeable SCC*mec* were resistant to at least three antibiotic classes, and some isolates presented with two or three recombinase complexes types, suggesting the presence of multiple SCC*mec* elements in tandem. However, to verify this, the *S. hominis* SCC*mec* cassette should be sequenced completely and compared to the *S. aureus* cassette. This analysis is currently underway in our laboratory.

We found that the 82% of *mecA*-positive isolates were untypeable and neither of the two methods used amplified any of the know recombinases suggesting that these strains are therefore likely candidates for carrying novel SCC*mec* types. This observation was previously described for *S. hominis* [11], [13], [21], [30], [33], [34] and may be explained by: a) that this cassette is a carrier of a new recombinase not related to *ccr*AB or *ccr*C genes, b) they represent new *ccr* complex isotypes that cannot be amplified by currently utilized *ccr* primers, or c) *ccr* genes were not present [23].

In this study, we identified a high rate of methicillin resistance (81%) in addition to resistance to other antibiotics among the clinical isolates studied; an observation previously reported for *S. hominis* and other CoNS species [17], [21]. All methicillin resistant isolates were also positive for SCC*mec* in addition to displaying resistance to most β-lactams antibiotics tested.

Among the *S. hominis* isolates collected in the present study none were clonal, therefore we concluded that infections caused by these isolates were not caused by dissemination of the same isolate throughout the hospital. Taking into account the fact that *S. hominis* is a component of the normal skin and mucous membrane flora, it is likely that these infections were endogenous.

In conclusion, our results showed that *S. hominis* is a biofilm producer and in combination with its high resistance rate to antibiotics, renders this species a serious threat for infections in immunocompromised patients. Finally, *S. hominis* isolates may possess different SCC*mec* types compared to those present in *S. aureus*.
